# Guanidinium tetra­bromidomercurate(II)

**DOI:** 10.1107/S1600536809005972

**Published:** 2009-02-25

**Authors:** Hiromitsu Terao, Thorsten M. Gesing, Hideta Ishihara, Yoshihiro Furukawa, B. Thimme Gowda

**Affiliations:** a Faculty of Integrated Arts and Sciences, Tokushima University, Minamijosanjima-cho, Tokushima 770-8502, Japan; b FB05 Kristallographie, Universität Bremen, Klagenfurther Strasse, 28359 Bremen, Germany; c Faculty of Culture and Education, Saga University, Saga 840-8502, Japan; d Graduate School of Education, Hiroshima University, Higashi-Hiroshima 739-8524, Japan; e Department of Chemistry, Mangalore University, Mangalagangotri 574 199, Mangalore, India

## Abstract

The Hg atoms in the crystal structure of the title compound, (CH_6_N_3_)_2_[HgBr_4_], are tetra­hedrally coordinated by four Br atoms and the resulting [HgBr_4_]^2−^ tetra­hedral ions are linked to the [C(NH_2_)_3_]^+^ ions by bromine–hydrogen bonds, forming a three-dimensional network. In the structure, the anions are located on special positions. The two different Hg⋯Br distances of 2.664 (1) and 2.559 (1) Å observed in the tetra­bromidomercurate unit are due to the connection of Br atoms to different number of H atoms.

## Related literature

For the ability of the guanidinium ion to make hydrogen bonds and its unique planar shape, see: Terao *et al.* (2000 ▶). For related literature, see: Ishihara *et al.* (2002 ▶); Furukawa *et al.* (2005 ▶)
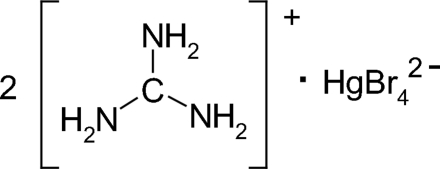


## Experimental

### 

#### Crystal data

(CH_6_N_3_)_2_[HgBr_4_]
*M* *_r_* = 640.41
Monoclinic, 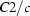

*a* = 10.035 (2) Å
*b* = 11.164 (2) Å
*c* = 13.358 (3) Å
β = 111.67 (3)°
*V* = 1390.7 (6) Å^3^
*Z* = 4
Mo *K*α radiation
μ = 22.53 mm^−1^
*T* = 298 K
0.09 × 0.09 × 0.09 mm

#### Data collection

Stoe IPDS-I diffractometer
Absorption correction: none
9651 measured reflections
1361 independent reflections
982 reflections with *I* > 2σ(*I*)
*R*_int_ = 0.093

#### Refinement

*R*[*F* ^2^ > 2σ(*F* ^2^)] = 0.030
*wR*(*F* ^2^) = 0.069
*S* = 0.90
1361 reflections
79 parameters
6 restraints
H atoms treated by a mixture of independent and constrained refinement
Δρ_max_ = 0.71 e Å^−3^
Δρ_min_ = −1.03 e Å^−3^

### 

Data collection: *EXPOSE* (Stoe & Cie, 1999 ▶); cell refinement: *CELL* (Stoe & Cie, 1999 ▶); data reduction: *XPREP* (Bruker, 2003 ▶); program(s) used to solve structure: *SHELXS86* (Sheldrick, 2008 ▶); program(s) used to refine structure: *SHELXL93* (Sheldrick, 2008 ▶); molecular graphics: *DIAMOND* (Crystal Impact, 2008 ▶); software used to prepare material for publication: *SHELXL93* (Sheldrick, 2008 ▶).

## Supplementary Material

Crystal structure: contains datablocks I, global. DOI: 10.1107/S1600536809005972/bt2874sup1.cif

Structure factors: contains datablocks I. DOI: 10.1107/S1600536809005972/bt2874Isup2.hkl

Additional supplementary materials:  crystallographic information; 3D view; checkCIF report

## Figures and Tables

**Table 1:** Hydrogen-bond geometry (Å, °)

*D*—H⋯*A*	*D*—H	H⋯*A*	*D*⋯*A*	*D*—H⋯*A*
N1—H1*A*⋯Br2^i^	0.87 (9)	3.03 (4)	3.845 (8)	158 (9)
N1—H1*B*⋯Br1^ii^	0.87 (9)	2.77 (6)	3.512 (7)	144 (8)
N2—H2*A*⋯Br1^iii^	0.87 (9)	2.72 (4)	3.541 (7)	159 (8)
N2—H2*B*⋯Br2^i^	0.87 (9)	2.74 (4)	3.535 (7)	153 (8)
N3—H3*A*⋯Br1^iv^	0.87 (9)	3.05 (10)	3.505 (8)	115 (8)
N3—H3*B*⋯Br1^iii^	0.87 (9)	2.98 (8)	3.667 (9)	137 (9)

Symmetry codes: (i) 
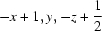
; (ii) 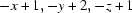; (iii) 
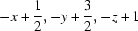
; (iv) 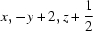.

**Fig. 1.**
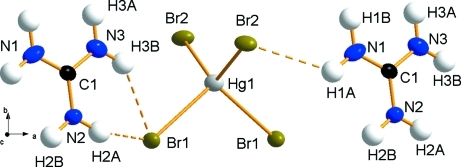
Molecular structure of (I), showing the atom labeling scheme. The displacement ellipsoids are drawn at the 50% probability level. The H atoms are represented as small spheres of arbitrary radii.

**Fig. 2.**
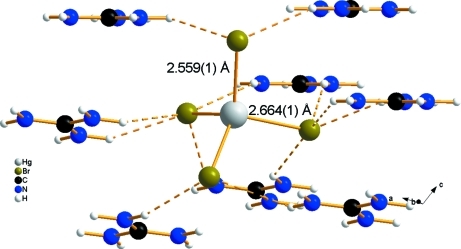
: Connection scheme of the HgBr42- tetrahedra with the [C(NH2)3]+ ions.

**Fig. 3.**
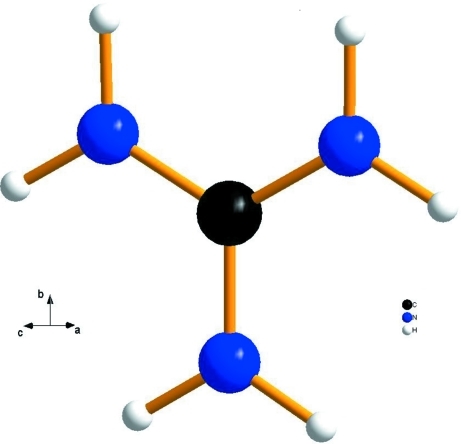
: The planar [C(NH2)3]+ ion.

## supplementary crystallographic information

### Comment

The guanidium ion, [C(NH_2_)_3_]^+^ is interesting due to its ability of making hydrogen bonds and its unique planar shape (Terao *et al.*, 2000). Further, the guanidium ions tend to undergo reorientation motions about their (pseudo) C_3_ axes in the crystals. Due to the soft nature of the Hg atom amenable to polarization, the Hg-halogen bonds are sensitive to the intermolecular interactions such as hydrogen bonding (Ishihara *et al.*, 2002). This was evident in the halogen NQR of the Hg compounds in which the resonance lines are widely spread in frequency (Furukawa *et al.*, 2005). Thus we are interested in studying the structure and bonding in this class of compounds. As a part of our study, we report herein the crystal structure of Guanidinium tetrabromidomercurate(II). In the structure, mercury atoms are tetrahedrally coordinated by four bromine atoms and the resulting HgBr_4_ tetrahedra are interconnected to the [C(NH_2_)_3_]^+^ ions by bromine-hydrogen bonds (Fig. 1) forming a three-dimensional network. In the tetrabromidomercurate unit, two different Hg—Br distances were observed: Hg—Br1 = 2.664 (1) Å and Hg—Br2 = 2.559 (1) Å. The shorter distance of the latter is due to its connection with two hydrogen atoms, whereas the Br1 is connected to four different hydrogen atoms, which elongate the Hg—Br bond (Fig.2). The C(NH_2_)_3_ moity (Fig. 3) itself is planar where the N—H bonds are somewhat elongated (1.01 (2) Å) to form the network bonds to the bromine atoms of the HgBr_4_ tetrahedra.

### Experimental

Guanidinium tetrabromidomercurate(II) was prepared by slow concentration of methanolic solution containing mercuric bromide (0.01 mole) and guanidium bromide (0.02 mole) in 1:2 molar ratio. The purity of the compound was checked by elemental analysis and characterized by its NMR and NQR spectra (Furukawa *et al.*, 2005). The single crystals used in X-ray diffraction studies were grown in methanolic solution by a slow evaporation at room temperature.

### Refinement

The N-H distances were restrained to 0.87 (1)Å and the coordinates of the  H atoms were refined with isotropic displacement parameters set to 1.2 times of the *U*_eq_ of the parent atom.

### Crystal data

**Table:**

(CH_6_N_3_)_2_[HgBr_4_]	*F*(000) = 1144
*M**_r_* = 640.41	*D*_x_ = 3.059 Mg m^−^^3^
Monoclinic, *C*2/*c*	Melting point: not measured K
Hall symbol: -C 2yc	Mo *K*α radiation, λ = 0.71073 Å
*a* = 10.035 (2) Å	Cell parameters from 2000 reflections
*b* = 11.164 (2) Å	θ = 2.9–26.1°
*c* = 13.358 (3) Å	µ = 22.53 mm^−^^1^
β = 111.67 (3)°	*T* = 298 K
*V* = 1390.7 (6) Å^3^	Cylindric, colourless transparent
*Z* = 4	0.09 × 0.09 × 0.09 mm

### Data collection

**Table:**

Stoe IPDS-I diffractometer	982 reflections with *I* > 2σ(*I*)
Radiation source: fine-focus sealed tube	*R*_int_ = 0.093
graphite	θ_max_ = 26.1°, θ_min_ = 2.9°
imaging plate dynamic profile intergration scans	*h* = −12→12
9651 measured reflections	*k* = −13→13
1361 independent reflections	*l* = −16→16

### Refinement

**Table:**

Refinement on *F*^2^	Secondary atom site location: difference Fourier map
Least-squares matrix: full	Hydrogen site location: inferred from neighbouring sites
*R*[*F*^2^ > 2σ(*F*^2^)] = 0.030	H atoms treated by a mixture of independent and constrained refinement
*wR*(*F*^2^) = 0.069	*w* = 1/[σ^2^(*F*_o_^2^) + (0.0376*P*)^2^] where *P* = (*F*_o_^2^ + 2*F*_c_^2^)/3
*S* = 0.90	(Δ/σ)_max_ = 0.001
1361 reflections	Δρ_max_ = 0.71 e Å^−^^3^
79 parameters	Δρ_min_ = −1.03 e Å^−^^3^
6 restraints	Extinction correction: *SHELXL93* (Sheldrick, 2008), Fc^*^=kFc[1+0.001xFc^2^λ^3^/sin(2θ)]^-1/4^
Primary atom site location: structure-invariant direct methods	Extinction coefficient: 0.00077 (10)

### Special details

**Table:**

Geometry. All esds (except the esd in the dihedral angle between two l.s. planes) are estimated using the full covariance matrix. The cell esds are taken into account individually in the estimation of esds in distances, angles and torsion angles; correlations between esds in cell parameters are only used when they are defined by crystal symmetry. An approximate (isotropic) treatment of cell esds is used for estimating esds involving l.s. planes.
Refinement. Refinement of *F*^2^ against ALL reflections. The weighted *R*-factor *wR* and goodness of fit *S* are based on *F*^2^, conventional *R*-factors *R* are based on *F*, with *F* set to zero for negative *F*^2^. The threshold expression of *F*^2^ > σ(*F*^2^) is used only for calculating *R*-factors(gt) *etc*. and is not relevant to the choice of reflections for refinement. *R*-factors based on *F*^2^ are statistically about twice as large as those based on *F*, and *R*- factors based on ALL data will be even larger.

### Fractional atomic coordinates and isotropic or equivalent isotropic displacement parameters (Å^2^)

**Table:**

	*x*	*y*	*z*	*U*_iso_*/*U*_eq_	
Hg1	0.5000	0.71191 (4)	0.2500	0.0577 (2)	
Br1	0.30789 (8)	0.86270 (7)	0.27353 (7)	0.0555 (2)	
Br2	0.38905 (10)	0.60032 (7)	0.07086 (6)	0.0635 (3)	
C1	0.4454 (8)	0.8215 (6)	0.6018 (6)	0.0492 (18)	
N1	0.5515 (10)	0.8736 (7)	0.5829 (6)	0.070 (2)	
H1A	0.592 (10)	0.818 (7)	0.558 (8)	0.084*	
H1B	0.549 (10)	0.950 (2)	0.594 (8)	0.084*	
N2	0.4254 (7)	0.7072 (6)	0.5904 (6)	0.0625 (17)	
H2A	0.363 (7)	0.673 (8)	0.612 (7)	0.075*	
H2B	0.485 (8)	0.665 (7)	0.571 (7)	0.075*	
N3	0.3560 (9)	0.8857 (7)	0.6335 (7)	0.075 (2)	
H3A	0.369 (11)	0.960 (3)	0.622 (8)	0.090*	
H3B	0.278 (7)	0.856 (9)	0.636 (9)	0.090*	

### Atomic displacement parameters (Å^2^)

**Table:**

	*U*^11^	*U*^22^	*U*^33^	*U*^12^	*U*^13^	*U*^23^
Hg1	0.0706 (3)	0.0544 (3)	0.0586 (3)	0.000	0.0359 (2)	0.000
Br1	0.0569 (4)	0.0485 (4)	0.0714 (5)	−0.0003 (3)	0.0359 (4)	−0.0070 (4)
Br2	0.0950 (6)	0.0453 (5)	0.0638 (5)	−0.0062 (4)	0.0454 (5)	−0.0100 (4)
C1	0.051 (4)	0.043 (4)	0.043 (4)	0.005 (3)	0.005 (3)	−0.006 (3)
N1	0.090 (5)	0.054 (4)	0.064 (5)	−0.021 (4)	0.026 (4)	−0.001 (4)
N2	0.058 (4)	0.052 (4)	0.081 (5)	−0.005 (3)	0.030 (4)	−0.011 (4)
N3	0.081 (5)	0.063 (5)	0.073 (5)	0.011 (5)	0.018 (5)	−0.009 (4)

### Geometric parameters (Å, °)

**Table:**

Hg1—Br2	2.5593 (10)	N1—H1A	0.87 (9)
Hg1—Br2^i^	2.5593 (10)	N1—H1B	0.87 (9)
Hg1—Br1	2.6639 (9)	N2—H2A	0.87 (9)
Hg1—Br1^i^	2.6639 (9)	N2—H2B	0.87 (9)
C1—N2	1.293 (10)	N3—H3A	0.87 (9)
C1—N1	1.316 (11)	N3—H3B	0.87 (9)
C1—N3	1.334 (11)		
			
Br2—Hg1—Br2^i^	121.74 (4)	C1—N1—H1A	107 (7)
Br2—Hg1—Br1	109.51 (4)	C1—N1—H1B	109 (7)
Br2^i^—Hg1—Br1	106.33 (3)	H1A—N1—H1B	144 (10)
Br2—Hg1—Br1^i^	106.33 (3)	C1—N2—H2A	120 (6)
Br2^i^—Hg1—Br1^i^	109.51 (4)	C1—N2—H2B	118 (7)
Br1—Hg1—Br1^i^	101.62 (4)	H2A—N2—H2B	121 (9)
N2—C1—N1	121.0 (8)	C1—N3—H3A	107 (8)
N2—C1—N3	118.3 (8)	C1—N3—H3B	122 (8)
N1—C1—N3	120.7 (7)	H3A—N3—H3B	125 (10)

Symmetry codes: (i) −*x*+1, *y*, −*z*+1/2.

### Hydrogen-bond geometry (Å, °)

**Table:**

*D*—H···*A*	*D*—H	H···*A*	*D*···*A*	*D*—H···*A*
N1—H1A···Br2^i^	0.87 (9)	3.03 (4)	3.845 (8)	158 (9)
N1—H1B···Br1^ii^	0.87 (9)	2.77 (6)	3.512 (7)	144 (8)
N2—H2A···Br1^iii^	0.87 (9)	2.72 (4)	3.541 (7)	159 (8)
N2—H2B···Br2^i^	0.87 (9)	2.74 (4)	3.535 (7)	153 (8)
N3—H3A···Br1^iv^	0.87 (9)	3.05 (10)	3.505 (8)	115 (8)
N3—H3B···Br1^iii^	0.87 (9)	2.98 (8)	3.667 (9)	137 (9)

Symmetry codes: (i) −*x*+1, *y*, −*z*+1/2; (ii) −*x*+1, −*y*+2, −*z*+1; (iii) −*x*+1/2, −*y*+3/2, −*z*+1; (iv) *x*, −*y*+2, *z*+1/2.
